# Langer-Giedion Syndrome: A Distinct Phenotype

**Published:** 2014-09-10

**Authors:** Riya George, Fehmida Najmuddin, Rajesh Rai, Keya Lahiri

**Affiliations:** 1 Department of Pediatrics, KJ Somaiya Medical College; 2 DY Patil Medical College, Hospital & Research Centre, India

**Keywords:** Trichorhinophalangeal Syndrome Type II, Exostoses, Microdeletion, Dysmorphic Features, Contiguous Gene Deletion Syndrome

Trichorhinophalangeal syndrome (TRPS), as the name suggests is a rare genetic disorder which affects the tricho (hair), rhino (nose), phalanges (digits)^[^^1^^]^ and has been classified into three types. Trichorhinophalangeal syndrome type II (TRPS2) is also known as Langer-Giedion syndrome (LGS). It was first described by Andreas Giedion, a Swiss pediatric radiologist and Leonard O Langer Jr, an American radiologist.

 TRPS2 combines features of trichorhinophalangeal syndrome type I (TRPS1) and multiple exostoses^[^^2^^]^. It is characterized by sparse hair, multiple cone shaped epiphyses, multiple cartilaginous exostoses, bulbous nasal tip, thickened alar cartilage, upturned nares, prominent philtrum, large protruding ears and mild mental retardation^[^^3^^]^. Multiple cartilaginous exostoses distinguishes TRPS2 from TRPS1.

 Exostoses are multiple projections of bone capped by cartilage, mostly seen in the metaphyses as well as diaphyses of long bones. Flat bones, vertebrae and the ribs may also be affected. As the bone continues to grow, the exostoses appear to migrate towards the diaphysis. At puberty as the growth plate fuses, the linear growth ceases and no new exostoses develop^[^^4^^]^. There are no trigger factors for development of exostoses.

 Microdeletions involve loss of small chromosome regions, the largest of which are detectable only with prophase chromosome studies or molecular methods. When such a deletion involves more than a single gene, the condition is referred to as a contiguous gene deletion syndrome^[^^5^^]^. TRPS2 is a true contiguous gene deletion syndrome with deletions in both TRPS1 and EXT1 genes on chromosome 8q24.1-q24.13.

 A 7 yr old female child born of a non-consanguineous marriage presented to a tertiary care hospital with failure to thrive and multiple cartilaginous exostoses. Mother had two first trimester abortions and two live healthy children. Child was immunized till date and was developmentally normal. 

**Fig. 1:**
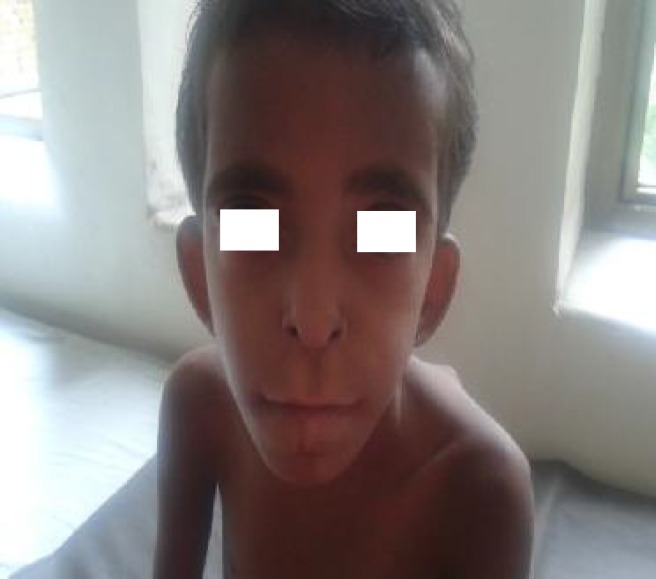
Classical dysmorphic features showing hypertelorism, bushy eyebrows, low set ears, large philtrum, prognathism, upturned nares

**Fig. 2:**
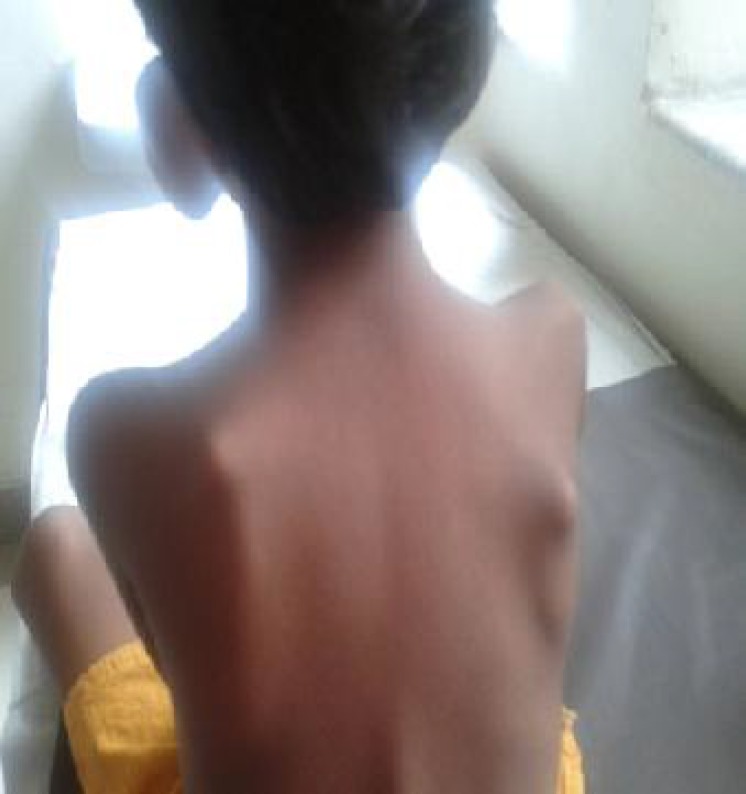
Exotoses over the scapulae

 On examination, her weight and height were below the 3rd percentile for age. Facial dysmorphism was noted which included microcephaly, sparse hair, bushy eyebrows, hypertelorism, long philtrum, micrognathia, high arched palate, poor dentition, low set ears, deformed ear cartilage and auricular sinus (Fig. 1). Limb deformities in the form of clinodactyly and overlapping of toes were observed. Multiple cartilaginous exostoses were noted over the ribs, elbows, wrists, knees and back (Fig. 2). Systemic examination revealed no abnormality.

**Fig. 3:**
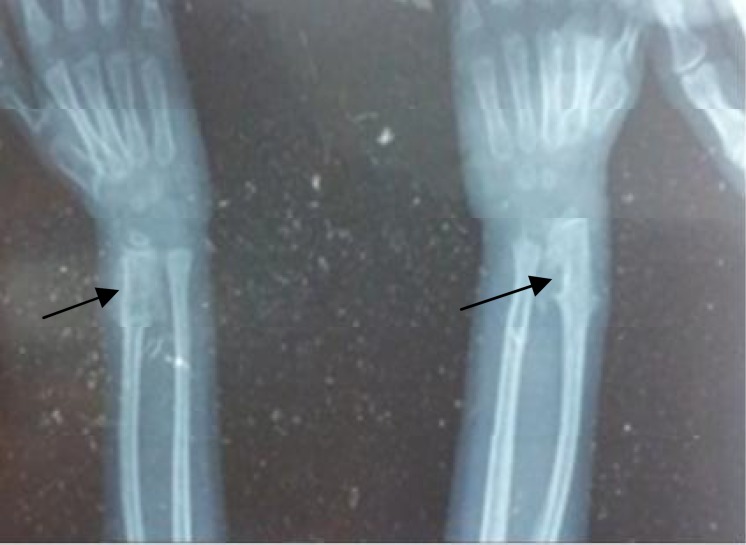
Radiograph of the wrist joint showing exostoses *crests*

 Her intelligence quotient evaluation was within normal range. Radiograph of the hands revealed delayed bone age, cone shaped epiphysis and exostoses bilaterally (Fig. 3). Chromosomal analysis in the form of karyotyping was done which was normal.

 These patients need a long-term follow-up throughout their life. Currently, our patient is 9 years old with normal intelligence and without any complications due to exostoses. The child is being followed-up monthly as these patients are known to develop complications such as Perthes disease, osteomas causing cervical nerve compression, growth hormone deficiency, infertility and malignant transformation^[^^6^^]^.

 Differential diagnosis includes Trichorhino-phalangeal syndrome type 1 which has similar dysmorphic features to Langer-Giedion syndrome but without exostoses. Others include metachondromatosis in which the exostoses are primarily seen in the hands and feet along with enchondromata in the ends of long bones and iliac crests. Most of the metachondromatoses regress spontaneously[7]. The other differential diagnosis is 11p11 deletion syndrome (OMIM 601224) which is also characterised by the presence of multiple exostoses but is differentiated from Langer-Giedion syndrome by the presence of cutaneous syndactyly, skull abnormalities like brachycephaly, turricephaly, enlarged parietal foramina and craniofacial dysostosis. 

 Treatment in Langer-Giedion syndrome is usually supportive but in case of complications due to exostoses such as pain, limited range of joint movement, pressure on nerves, blood vessels, the spinal cord, and tissues, surgical intervention is necessary. 

 To conclude, our case is an unusual one, as the child presented as a classical LGS phenotype with multiple exostoses and typical dysmorphic features but without a gene deletion in the *TRPS1* and *EXT1 *genes. (A similar case was presented by Pereza et al, 2012)^[^^8^^]^
